# P38 The AMR Patient Shield (APS) project: patient empowerment in the fight against antimicrobial resistance

**DOI:** 10.1093/jacamr/dlad066.042

**Published:** 2023-06-14

**Authors:** Aman Gupta, Dele Famokunwa, Tom Ashfield, James Amos, Mineli Cooray

**Affiliations:** Pfizer Ltd; Pfizer Ltd; Pfizer Ltd; Pfizer Ltd; Pfizer Ltd

## Abstract

**Background:**

Pfizer and the Patient’s Association (PA) collaborated on the AMR Patient Shield project, which aimed to explore the role of patients in responding to antimicrobial resistance (AMR) and empower them to work with their clinicians to support appropriate use of antibiotics in their care. The project had two phases, with a focus on engagement and education of patients, culture change and education of healthcare professionals (HCPs).

**Methods:**

In Phase I, patients were convened for a patient advisory board in 2021, and Phase II involved two subsequent resource co-creation workshops in 2022. PA recruited nine patients for each phase, and the meetings were co-facilitated by PA and Pfizer medical representatives. Two antimicrobial pharmacists also served as advisors in Phase II to provide input on the applicability of the proposed resources within the clinical setting.

**Outcomes:**

The project resulted in a range of tailored resources for both patients and HCPs, aimed at supporting patients to also become stewards of appropriate antibiotic use. These resources included educational leaflets, a template patient-held microbiology record, an HCP antibiotic consent checklist, and audio-visual content. The resources developed for patients improved their literacy on AMR at both the population and individual health levels, enabling them to utilize the incorporated tools for shared decision-making conversations with their HCPs. The content designed specifically for HCPs aimed to improve their ability to obtain informed consent, maintain accurate records and support clinical decision making when prescribing antimicrobials.

**Conclusions:**

The AMR Patient Shield project highlights the shift from working *for* patients to working *with* patients, emphasizing the vital importance of shared decision making between HCPs, industry, policy makers and patients in the stewardship agenda. By involving patients as active partners in the AMR response strategies, the project aims to empower them to participate in their own care, improve their understanding of AMR and engage in SDM discussions with their clinicians. In conclusion, the AMR Patient Shield project, a collaboration between Pfizer and the Patient’s Association, has resulted in tailored resources for patients and HCPs to support their engagement in appropriate antibiotic use. These resources aim to improve patient literacy on AMR and enhance HCPs’ ability to obtain informed consent and exercise clinical judgement. The project highlights the importance of shared decision making and collaboration among stakeholders in addressing AMR, placing patients as fundamental partners in the collective response to this global health challenge.

